# Telephonic intervention to combat non-adherence to oral iron-folic acid supplementation in pregnancy: A randomized controlled trial

**DOI:** 10.1016/j.eurox.2023.100235

**Published:** 2023-09-09

**Authors:** Sakshi Sharma, M.V. Smitha, Deepthy Balakrishnan

**Affiliations:** a College of Nursing, All India Institute of Medical Sciences, Bhubaneswar, Odisha 751019, India; b Department of Obstetrics and Gynecology, All India Institute of Medical Sciences, Bhubaneswar, Odisha 751019, India; c All India Institute of Medical Sciences, Gorakhpur, Uttar Pradesh 273008, India

**Keywords:** Counseling, Medication therapy management, Patient compliance, Telemedicine

## Abstract

**Introduction:**

Iron deficiency anemia is a public health problem globally attributing to high incidences of maternal and infant mortality and morbidity. Iron and folic acid supplementation (IFAS) is essential and provided free of cost by the public health sectors, however, a systematic review shows that the national-level adherence to oral Iron-Folic Acid Supplementation (IFAS) is less than half in pregnant women, and the significant obstacles to non-adherence are fear of side effects and forgetfulness. This trial was designed to mitigate the side effects and tackle forgetfulness with telephonic intervention. The objectives were to investigate the effectiveness of the telephonic intervention on oral IFAS adherence and hemoglobin and the reasons for non-adherence to oral IFAS, to find out the proportion of anemia in the study population, and to assess the effectiveness of the intervention on maternal and neonatal outcomes.

**Methods:**

Hospital-based open-label multi-centric parallel-group randomized controlled trial, used block randomization and allocated treatment in a 1:1 ratio recruited 286 anemic pregnant women between 14 and 24 weeks of gestation with hemoglobin level < 11 g/dl having smartphones at a secondary hospital and a tertiary hospital in Eastern India. The experimental group received telephonic intervention for one month via structured text reminders, WhatsApp audio messages, and phone calls. The standard course of treatment was given to the control group.

**Results:**

286 women (n_1_ =143, n_2_ =143) were randomized, 36 had attrition leaving 250 for analysis (n_1_ =123, n_2_ =127), the experimental group experienced a 44.9 % and the control group 13.8 % increase in adherence (*P* < 0.001). The leading reasons for non-adherence were forgetfulness (24 %), nausea and vomiting (23.2 %), and constipation (18.8 %). Hemoglobin level increased by 0.8 g/dl (*P* < 0.001) in the experimental group and 0.2 g/dl (*P* < 0.807) in the control group.

**Conclusion:**

In addition to improving adherence to oral IFAS, telephonic intervention mitigates side effects and enhances hemoglobin in anemic pregnant women. The increase in adherence was threefold in the experimental group compared to a marginal rise in the control group. This study recommends the implementation of a telephonic intervention to promote adherence to oral IFAS among anemic pregnant women.

## Highlights

• Poor adherence to oral IFAS in pregnancy increases the risk of complications.
• Poor adherence is a chief reasons for the persistent high prevalence of anemia.
• Telephonic intervention is a client-friendly approach to boost IFA compliance and mitigate the side effects.

## Graphical Abstract

**Figure:**
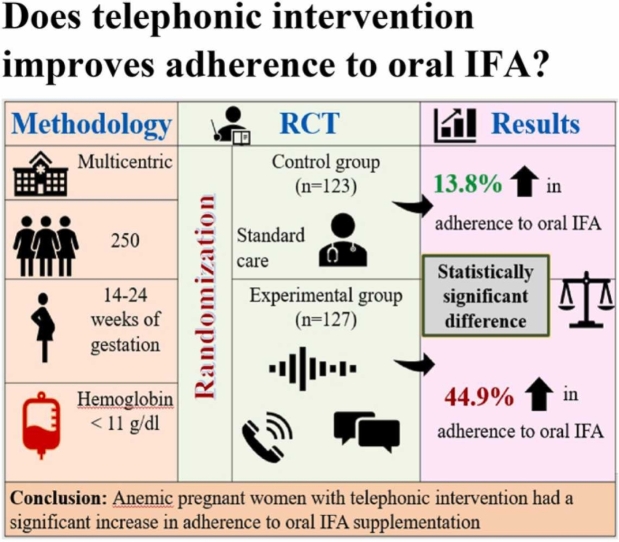

## 1 Introduction

A significant contributing factor to the high Maternal Mortality Rate (MMR) is anemia, and iron deficiency anemia has detrimental consequences on the health of pregnant women and newborns [1], [2] According to the WHO, anemia is blood hemoglobin levels less than 11 g/dl in pregnant women [3]. In Southeast Asia, 52 % of women of childbearing age are affected [4]. Anemic pregnant women aged 15–49 account for 52.2 % in India, whereas 61.8 % in Odisha, only 34.4 % have taken 180 or more oral Iron-Folic Acid Supplementation (IFAS). (NFHS-5).

Nutrition deficiencies are common, and iron is the most deficient micronutrient [5]. Daily oral IFAS during pregnancy reduces the 70 % risk of iron deficiency anemia at term [5]. Only one-third of pregnant women had desirable adherence to their common antenatal drugs [6]. Low Antenatal Care (ANC) services, insufficient availability, poor counseling, and lack of information about anemia and its impact contribute to poor IFAS adherence worsened by the COVID-19 pandemic. [5].

Several methods to measure medication adherence are available, including direct and indirect. Direct methods comprise observation by Health Care Workers (HCWs) and pill counts and are effective [7], but in a resource-poor setting with a workforce shortage, this approach is not practically applicable in delivering ANC services. Indirect methods include patient interviews and lab reports. Although newer technology for assessing medicine adherence, such as electronic monitoring, has shown promising effects, much of it is yet to be tested in well-controlled clinical studies [8].

More efforts are required to meet the World Health Assembly’s target of a 50 % reduction in anemia prevalence in the reproductive age group by 2025 [9]. Digital technology is critical in accelerating the attainment of the SDGs by 2030 and ensuring that no one is left behind [10]. The world’s population comprises more than seven billion mobile subscribers, and mobile services have covered 95 % till December 2018. Digitalization and mHealth have a chance to expand and change how we obtain health care. They can address people in rural areas and make universal health care a reality worldwide [11] Telephonic health counseling has shown to be a simple and effective alternative as the calls can be scheduled. An attempt has been made to use pregnant women’s mobile phones to build awareness and management of anemia, where counseling can be provided in a secure atmosphere without intruding the privacy [12].

This novel telephonic intervention was designed to mitigate the side effects and tackle forgetfulness of oral IFAS during pregnancy. The objectives were to investigate the effectiveness of the telephonic intervention on oral IFAS adherence and hemoglobin levels and to identify the reasons for non-adherence.

## 2 Materials and methods

### 2.1 Trial design and study setting

This hospital-based open-label parallel-group randomized controlled trial was conducted in Obstetrics and Gynecology (OBG) OPD of secondary and tertiary care centers. The recruitment started on 1st October 2021, and the follow-up was completed on 29th April 2022. The intervention was performed in compliance with the relevant laws, and approval was obtained from the Institutional Ethical Committee (IEC/AIIMS BBSR/Nursing/2021-22/05), CDMO of the District, and prospectively registered in the Clinical Trials Registry (CTRI/2021/09/036603). Informed written consent was obtained from the participants.

### 2.2 Participants

**Fig. 1:**
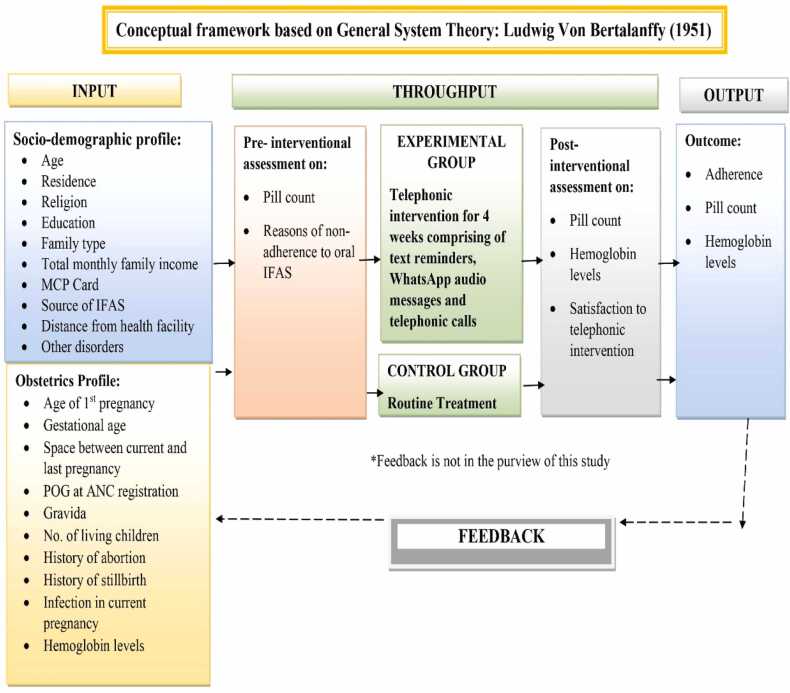
Conceptual framework of the study.

A total of 286 anemic pregnant women were recruited. The study sample was calculated using the GPower 3.1 software (0.45 effect size from the pilot study), 5 % level of significance, 80 % power, 30 % attrition, and 143 were taken as the final sample in each group. Inclusion criteria had pregnant women with a smartphone of their own or husband with hemoglobin less than 11 g/dl between 14 and 24 weeks of gestation receiving ANC services and willing to participate in the study. Women with a known case of psychiatric disorder were excluded from the study. The conceptual framework was designed based on General System Theory by Ludwig Von Bertalanffy (1951) (Fig. 1).

**Fig. 2:**
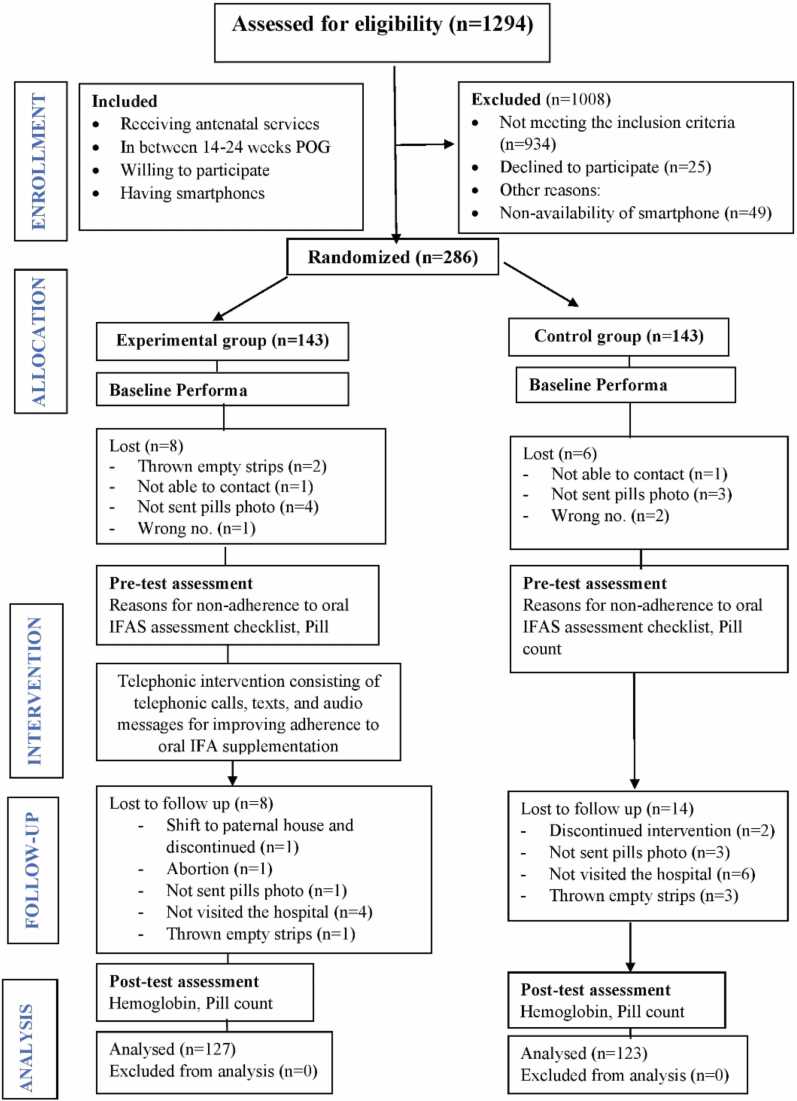
CONSORT flow diagram of the study.

The attrition rate was 13.2 %. The analysis was performed on 127 women in the experimental and 123 women in the control group. The detailed CONSORT flow diagram is given in Fig. 2.

Oral IFAS was used as per the government guidelines (60 mg elemental iron and 0.5 mg folic acid). Adherence refers to the consumption of all the prescribed pills of oral IFAS estimated by pill count.

### 2.3 Randomization and masking

Block randomization was done using blocks 4 and 6; the third party generated the randomization sequence using the online site. [13] Allocation concealment was done through sealed, opaque envelopes which have C or E written on the paper kept inside as the control and experimental group codes, respectively. According to the code, the women picked up the envelope, the allocation was done in 1:1, and 143 women were recruited in each group. Considering the nature of the intervention, blinding could not be done. However, the laboratory technicians estimating the hemoglobin levels were unaware of the allocation.

The tools were found to be valid and reliable and were translated to the Odia language and back-translated to the English language by two independent language experts and were pre-tested on 20 women.

### 2.4 Intervention

**Fig. 3:**
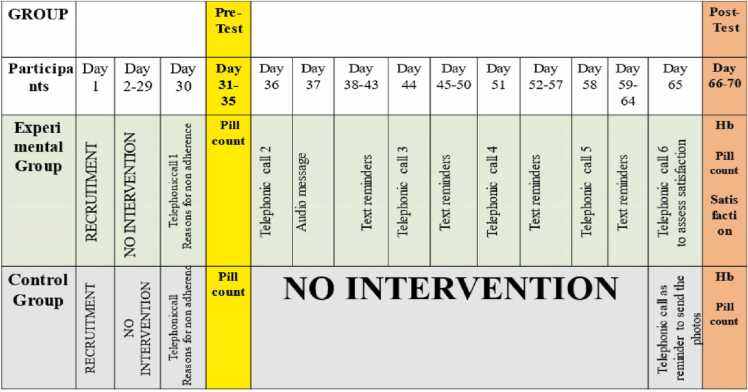
Schematic representation of the Intervention protocol.

The intervention was specifically tailored to meet the needs of the study population. The content was validated by various experts. The intervention was provided only after achieving the competence and training from a registered counselor. The women in the control group received conventional treatment, while the experimental group received telephonic intervention. The schematic representation of the intervention protocol is shown in Fig. 3. The detailed intervention can be accessed on request.

### 2.5 Outcomes

The study’s primary outcome was adherence to oral IFAS. The secondary outcomes were the reasons for oral IFAS non-adherence, hemoglobin levels, the proportion of anemia, satisfaction with the telephonic intervention, and maternal and neonatal outcomes.

### 2.6 Statistical analysis

Analysis was performed using IBM SPSS Statistics for Windows, Version 20 (IBM Corp. NY, USA). Specific tests were applied based on the data distribution, and the KS test was used to check for the normality of the data. Descriptive and inferential statistics were used to analyze the data.

## 3 Results

### 3.1 Primary outcomes

Out of 1249 eligible women, 1008 were excluded, and 286 were randomized. Both the groups were homogenous and comparable at baseline, and a *P* < 0.05 was considered significant. (Table 1) The effectiveness of the telephonic intervention on adherence to oral IFAS was compared within the groups using the Mc Nemar test and between the groups using the Chi-square test. (Table 2) The Mc Nemar test shows that within the group adherence to oral IFAS in the control group is statistically significant compared to during the pre-test and post-test. (z = 45.834, ***P***<0.001). In the control group, the adherence in the pre-test was 25.2 % and it increased to 39 % after one month. Mc Nemar test shows that within the group the adherence to oral IFAS in the experimental group is statistically significant compared to during the pre-test and post-test (z = 7.208, ***P***<0.007). The adherence in the experimental group before the intervention was 18.9 % whereas after the intervention it increased to 63.8 %. Between the group, the chi-square test shows that the difference in adherence to oral IFAS is more in the experimental group as compared to the control group which is statistically significant (z = 15.332, ***P***<0.001).

**Table 1:** Distribution of sociodemographic and obstetric variables.

n = 250					
Variables	Control group (n_1_=123)	Experimental group (n_2_=127)	df	t	*P* value
**Age (yr), mean+SD**	24.8 (4.1)	24.3 (3.9)	248	-0.853^a^	0.395
**Age at 1**^**st**^**pregnancy (yr), mean+SD**	22.7 (3.4)	22.3 (3.3)	248	-0.886^a^	0.376
**Residence**					
Rural	116 (94.3)	118 (92.9)	1	0.203^c^	0.
Urban	7 (5.7)	9 (7.1)			
**Religion**					
Hindu	116 (94.3)	113 (89)			
Muslim	6 (4.9)	12 (9.4)	2	2.354^b^	0.315
Christian	1 (0.8)	2 (1.6)			
**Education**					
Graduate and above	19 (15.4)	20 (15.7)			
Secondary school	29 (23.6)	19 (15)	3	5.272^b^	0.129
Primary school	75 (61)	85 (66.9)			
No formal education	-	3 (2.4)			
**Family type**					
Nuclear	17 (13.8)	18 (14.2)	1	0.006^c^	0.936
Joint	106 (86.2)	109 (85.8)			
**Total monthly family income (rupees)**					
**<**10,000	59 (48)	53 (41.7)	2	1.032^c^	0.597
10,000–30,000	52 (42.3)	59 (46.5)			
> 30,000	12 (9.7)	15 (11.8)			
**MCP card**					
Yes	108 (87.8)	99 (78)	1	4.259^c^	0.039
No	15 (12.2)	28 (22)			
**Source of IFA**					
Government supply	116 (94.3)	120 (94.5)	1	0.004^b^	0.951
Self- purchase	7 (5.7)	7 (5.5)			
**Distance from a health facility (km)**					
<10	27 (21.9)	36 (28.4)			
10–29	58 (47.2)	60 (47.2)	3	2.451^c^	0.484
30–49	33 (26.8)	25 (19.7)			
> 50	5 (4.1)	6 (4.7)			
**Gestational age (weeks)**					
14–17	40 (32.5)	49 (38.6)	2	6.938^c^	0.031
18–20	33 (26.8)	46 (36.2)			
21–24	50 (40.7)	32 (25.2)			
**POG at ANC registration (weeks)**					
<10	37 (30.1)	44 (34.6)			
10–12	37 (30.1)	49 (38.6)	3	5.929^c^	0.115
13–18	34 (27.6)	27 (21.3)			
> 18	15 (12.2)	7 (5.5)			
**No. of living children**					
0	68 (55.3)	92 (72.4)	2	7.984^c^	0.018
1	47 (38.2)	30 (23.6)			
> 2	8 (6.5	5 (3.9)			
**History of abortion**					
Yes	13 (10.6)	22 (17.3)	1	2.367^c^	0.124
No	110 (89.4)	105 (82.7)			
**History of stillbirth**					
Yes	1 (0.8)	2 (1.6)	1	0.035^b^	<0.999
No	122 (99.2)	125 (98.4)			
**History of infection in the current pregnancy**					
Yes	6 (4.9)	5 (3.9)	1	0.132^c^	0.717
No	117 (95.1)	122 (96.1)			
**Gravida**					
Primi	64 (52)	75 (59.1)	1	1.248^c^	0.264
Multi	59 (48)	52 (40.9)			

^a^Independent sample t-test, ^b^-Fisher’s exact test, ^c^-Chi-square test; SD, Standard Deviation; MCP card, Mother and child protection card; POG, Period of Gestation; ANC, Antenatal care.

**Table 2:** Comparison of oral IFAS adherence within and between the groups.

n = 250					
Group		Pretest	Post-test	Within the group Mc Nemar test z	*P* value
		n (%)	n (%)		
**Control** **(n**_**1**_**=123)**	Adherence	31 (25.2)	48 (39)		
	Non-adherence	92 (74.8)	75 (61)	45.834	<0.001*
**Experimental** **(n**_**2**_**=127)**	Adherence	24 (18.9)	81 (63.8)	7.208	0.007*
	Non-adherence	103 (81.1)	46 (36.2)		
**Between the group Chi-square test** **(ꭓ**^**2**^**)**		1.448	15.332		
***P*****value**		0.229	<0.001*		

Between the group, Chi-square test; within the group, Mc Nemar test; *P* * **<0.001 compared to control in between the group and pretest within the group

Both the groups demonstrated a statistically significant change in adherence to oral IFAS following the intervention, with the experimental group having a 44.9 % increase and a 13.8 % increase in the control group. With a net increase of 31.1 % in the experimental group over the control group.

### 3.2 Secondary outcomes

The most common reasons for non-adherence to oral IFAS were forgetfulness (24 %), nausea, and vomiting (23.2 %), followed by constipation (18.8 %) (Table 3).

**Table 3:** Distribution of the reasons for non-adherence to oral IFAS.

n = 250		
Reasons for non-adherence	n	%
Forgetfulness	60	24
Nausea and vomiting	58	23.2
Constipation	47	18.8
Gastric irritation	46	18.4
Keeping at a place where it was not visible	43	17.2
Gas formation	38	15.2
Household chores	30	12
Do not want to take the tablet	30	12
Birth of big baby	28	11.2
Dislike smell	25	10
Heartburn	24	9.6
Black stools	24	9.6
Fasting	20	8
Fear of interaction	20	8
The tablet will not increase iron in the blood	19	7.6
Complicate pregnancy	18	7.2
Dislike taste	18	7.2
Difficult delivery	17	6.8
Lack of support	16	6.4
Unavailability in pharmacy	16	6.4
Harmful for baby	15	6
Tablets should not be taken during pregnancy	15	6
Family functions	15	6
Inconvenient dosing schedule	14	5.6
Consumption of too many tablets	13	5.2
Fear of asking for a lost prescription	11	4.4
Traveling	9	3.6
Inadequately explained prescriptions by HCWs	8	3.2
Financial burden	8	3.2
Job commitments	7	2.8

HCWs, Health Care Workers.

The effectiveness of the intervention on hemoglobin levels was compared by the median and IQR using the Mann-Whitney U test and was found to have a statistically significant difference between the groups after the intervention and before the delivery (Table 4).

**Table 4:** Median and IQR of hemoglobin levels.

n = 250					
Variable		Control group (n_1_=123) Median (IQR)	Experimental group (n_2_=127) Median (IQR)	t	*P* value
**Hemoglobin levels (g/dl)**	Baseline	10 (9.4–10.4)	10 (9.2–10.6)	-0.244	0.807
	After intervention	10.2 (9.8–10.6)	10.8 (10.2–11.1)	-5.365	<0.001***
	Before delivery	10.3 (10–10.9)	10.7 (10.1–11.2)	-2.950	0.003*

Mann Whitney U test, P * ** < 0.001 compared to the control group; values are shown in median (IQR).

**Fig. 4:**
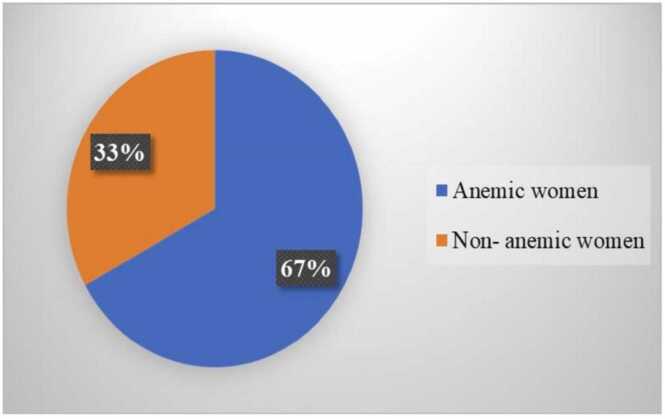
Proportion of anemia among pregnant women.

The proportion of anemia in the study population was found to be 67 %. (Fig. 4) The maternal and neonatal outcomes were followed and analyzed using the Mann-Whitney U test and Chi-square test. The mean birth weight of newborns was 2614.5+ 306.4 g in the control group, whereas 2671.6+ 353.7 g in the experimental group; there was no statistically significant difference between the groups (Table 5).

**Table 5:** Distribution of maternal and neonatal outcomes.

n = 250				
Variables	Control group (n_1_=123)	Experimental group (n_2_=127)	t	*P* value
**Birth weight (g), mean+SD**	2614.5 (306.4)	2671.6 (353.7)	-1.491^a^	0.136
**APGAR score (At 1 min), mean+SD**	8.8 (0.4)	8.9 (0.3)	-0.579^a^	0.562
**POG at delivery (days), mean+SD**	266 (12)	269 (13)	-1.835^a^	0.066
**Birth outcome**				
Live birth	122 (99.3)	126 (99.2)	0.001^c^	0.982
IUD	1 (0.8)	1 (0.8)		
**Mode of delivery**				
SVD	79 (64.2)	72 (56.7)	1.483^c^	0.223
LSCS	44 (35.8)	55 (43.3)		
**NICU admission**				
Yes	8 (6.5)	4 (3.1)	0.176^c^	0.675
No	115 (93.5)	123 (96.9)		
**PPH**				
Yes	3 (2.4)	1 (0.8)	1.083^c^	0.298
No	120 (97.6)	126 (99.2)		

^a^-Mann Whitney U test, ^c^-Chi-square test; SD, Standard Deviation; POG, Period of Gestation; IUD, Intrauterine Death; SVD, Spontaneous Vaginal Delivery; LSCS, Lower Segment Cesarean Section; NICU, Neonatal Intensive Care Unit; PPH, Post-Partum Hemorrhage.

## 4 Discussion

This study was intended to compare the effectiveness of the telephonic intervention on adherence to oral IFAS, hemoglobin levels and, identify the causes of non-adherence, find out the proportion of anemia in the study population. Oral IFAS is brushed aside by roughly two-thirds of pregnant women. The findings indicate that adherence improved by 44.9 % in the experimental group and 13.8 % in the control group. Some factors such as the women’s own interest in the management of anemia, the impact of social media, family members and friends, and services provided by ASHA workers and the obstetrician over a time period might have an impact and these could be the probable reasons for the increase in adherence in the control group. The same would have happened in the experimental group as well. That is why the design of the study had a control group to compare and monitor any changes after the telephonic intervention. Both the groups were homogeneous i.e. having similar sociodemographic and obstetrics characteristics were comparable at baseline before the start of the intervention. Hence, the higher increase in adherence by 44.9 % in the experimental group and 13.8 % control group can be justified.

The main reason for non-adherence was forgetfulness (24 %), and the increase in hemoglobin levels in the experimental group was 0.8 g/dl and 0.2 g/dl in the control group. This difference might be because of the impact of individual-centered comprehensive antenatal care, facilitating home-based management by optimal resource utilization and reducing geographical differences.

The current study revealed that 25.2 % in the experimental group and 18.9 % in the control group consumed all the IFAS prescribed, which is less than the study conducted in the urban area of south India. The probable reason for this difference could be that only 24.2 % of women were anemic. In contrast, the present study only considered anemic pregnant women as a population which suggests that the anemic status could be because of the low consumption of IFAS [14]. But adherence was lower than in the studies conducted in different regions of Ethiopia, ranging from 44 % to 63.6% [5], [15], [16]. This may be due to defining adherence as consumption of 65 % oral IFAS. In contrast, in the present study, 100 % consumption of the prescribed oral IFAS by anemic pregnant women in a month was operationally defined as adherence. Higher adherence was reported in studies conducted in Puducherry (63.8 %) and Sudan (92.1 %) [17], [18]. This might be because these studies were conducted at tertiary institutions that offer adequate counseling and continuous product availability. Moreover, this cannot be considered as the actual scenario in the community. At the same time, the present study involved most women from the district hospital serving the local community.

The present study revealed that telephonic intervention was one of the reasons for improved adherence to oral IFAS. It unleashed opportunities and bridged the gap in public health by focusing on the need-based mitigation of side effects of oral IFAS. Similar results were seen in an interventional study conducted in Ethiopia [19]. The current study revealed a three-fold increase in adherence to oral IFAS in the experimental group, whereas there was a marginal increase in the control group. Research in Kenya found that there was no statistically significant change in compliance in the experimental and control groups [20]. It could be because of the high attrition rate due to strikes among healthcare personnel, disrupting healthcare services. There was an increase of 43 % compliance in the experimental group over and above the control group. This could be due to direct observations and home visits [21]. Direct observation of oral IFAS among pregnant women in Haryana was statistically significant in increasing compliance (*P* = 0.001) [7]. However, it is not feasible to afford a direct contact intervention strategy for the workforce and finances in a resource-poor setting like ours.

The present study considered factors such as the shortage of HCWs for effective counseling and designed a telephonic intervention to provide efficient ANC and enhance women’s satisfaction. Insufficient counseling is a barrier to adherence [22]. Low awareness about the consequences of anemia, negative beliefs, lack of reminders, management of side effects caused by oral IFAS, and follow-up mechanisms were significant reasons for non-adherence [23]. Healthcare professionals provided conflicting information about oral IFAS to pregnant women, and it was inconsistent and omitted many details, such as the description and mitigation of side effects [24].

The current study showed that the most common reason for non-adherence was forgetfulness (24 %). These findings were like studies conducted in Mangalore [14], Puducherry 32 % [17], Nigeria 27.2 % [26], Southern Ethiopia [15], and West Iran [27]. Studies reported a very high forgetfulness rate in India (60 %) [7] and Africa (70.1 %) [25]. During the antenatal visit, good quality counseling should address forgetfulness. Designing strategies for reminding women to take their medicines on time is essential such as placing the tablets on a site they see every day. Interventions that provide adequate awareness, reminders, and text messages may help address the problem [26], [27]. Present study reported that the second most common reason for non-adherence was side effects. This was concordant with the results of other studies conducted elsewhere [14], [15], [17], [27], [28]. The most common side effect reported in this study was nausea and vomiting (23.2 %), followed by constipation 18.8 % and gastric irritation 18.4 %. A study from Mangalore too reported 21.54 % constipation and gastritis among 13.84 % of women [14]. Another study reported similar findings, 25.7 % [26], and higher rates of vomiting, 47.69 % [14]. The present study reported that 12 % of women do not want to consume oral IFAS [17]. This might be due to the discomfort caused by oral IFAS. A study found that flavor coating the pills and reducing the size and number of dosages could enhance adherence to oral IFAS [17]. Myths and misconceptions affect adherence to oral IFAS. In the present study, women believe that it will cause complications during pregnancy (7.2 %), and the birth of a big baby leads to cesarean section (11.2 %). Another study reported slightly fewer antenatal complications (2.2 %) and fear of having a big baby (7.2 %) [26].

The present study included one month of intervention and revealed a 0.6 g/dl increase in hemoglobin levels in the experimental group over and above the control group. These findings were concordant with a study from Haryana where the experimental group’s mean hemoglobin levels increased by 0.52 g/dl above the control group (*P* < 0.001) [7]. This might be because the utilization of ASHAs for a weekly supervised dose of IFAS helped improve compliance by overcoming side effects and reducing forgetfulness among pregnant women. However, another study reported a higher increase of 1.13 g/dl; this could be due to the long intervention period of 12 weeks [21]. The study findings can be generalized to similar setting comprising similar population.

### 4.1 Study limitations

In this trial, the effectiveness of the information provided by HCWs while prescribing oral IFAS was not assessed. Cluster randomization would be a preferable option to reduce the risk of contamination. The pill count may be affected by record bias and social desirability bias. Women might have attended private clinics and received different brands of oral IFAS, which was not explored. By emptying the pill strip without consuming the supplements, women may have sent pics of the pills. Only the hemoglobin levels were used to identify whether a woman had an iron deficit; additional indicators such as serum ferritin, total iron-binding capacity, folic acid level, and hematocrit were not examined because of financial restrictions. Due to the different hemoglobin estimation techniques used in the two settings, there may be a slight fluctuation in the hemoglobin levels.

### 4.2 Recommendations

The evil of anemia might be combated by developing platforms such as mobile applications, smart user interfaces, and digital dispensaries to provide obstetric services. Establishing midwife-led clinics and creating a recruitable post for tracking pregnant women, and scheduling system-generated customized messages, reminders, and calls. Encouraging the creation of a robust digital ecosystem focusing on adherence to daily oral IFAS, eliminating common misconceptions, management of side effects, communicating the advantages of oral IFAS for women and fetuses, and joining hands together for a technological revolution to defeat anemia. Strengthening the digital skills of the HCWs by including digital health in the curriculum of students. These strategies might help reduce the prevalence of iron-deficiency anemia in pregnant women.

## 5 Conclusion

Although iron deficiency anemia can be easily managed throughout pregnancy, it remains an unmet health need. A telephonic intervention might be implemented to promote adherence to oral IFAS among pregnant women. During antenatal appointments, health practitioners must present all women with health information regarding the benefits of oral IFAS. .

**Fig. 5:**
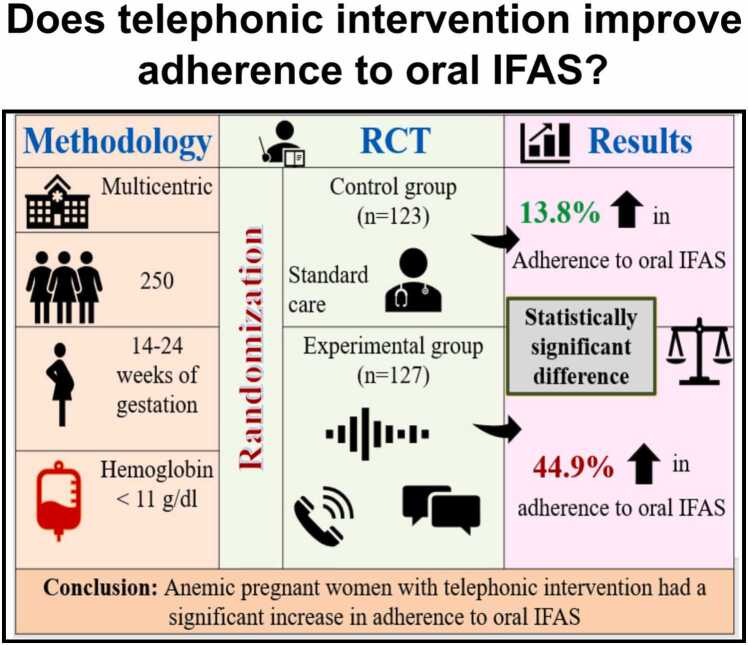
Does telephonic intervention improves adherence to oral IFA?.

## Ethical issues

None to be declared.

## Funding

Nil.

## Declaration of Competing Interest

The authors declare that they have no known competing financial interests or personal relationships that could have appeared to influence the work reported in this paper.

## Data Availability

The datasets generated during and analyzed during the current study are available from the corresponding author upon reasonable request.
